# Correction: Genetic analysis of *IFNG-AS1* implicates opposite effects to *Leishmania guyanensis*-cutaneous leishmaniasis: rs4913269 confers protection while rs7134599 enhances susceptibility and correlates with high plasma IL-4 and IL-10 levels

**DOI:** 10.1371/journal.pntd.0013656

**Published:** 2025-10-30

**Authors:** Marcus Vinitius de Farias Guerra, Josué Lacerda de Souza, Lener Santos da Silva, José do Espírito Santo Júnior, Tirza Gabrielle Ramos de Mesquita, Krys Layane Guimarães, Duarte Queiroz, George Allan Villarouco da Silva, Mauricio Morishi Ogusku, Mara Lúcia Gomes de Souza, José Pereira de Moura Neto, Aya Sadahiro, Rajendranath Ramasawmy

There are errors in the Funding statement. The correct Funding statement is as follows: This work was supported by the Brazilian Council for Scientific and Technological Development (CNPq), grant number 404181/2012-0 to RR, Fundação de Amparo e Pesquisa do Estado do Amazonas (FAPEAM), grant numbers 06200151/2020, 01.02.016301.03393/2021-80 and 01.02.016301.01090/2023-94 to RR, and FAPEAM EDITAL N. 038/2022-PDGD/CAPES/FAPEAM – Coordenador/Auxilio Financeiro. JJ, JS, LS and KLGDQ have fellowships from FAPEAM. JPN and RR are CNPq fellows. The funders had no role in study design, data collection and analysis, decision to publish, or preparation of the manuscript.
