# Supplementary material for: Genetic analysis of IFNG-AS1 implicates opposite effects to Leishmania guyanensis-cutaneous leishmaniasis: rs4913269 confers protection while rs7134599 enhances susceptibility and correlates with high plasma IL-4 and IL-10 levels
Source: PLoS Negl Trop Dis. 2025 Jul 14;19(7):e0013318. doi: 10.1371/journal.pntd.0013318 (PMC12273940; doi:10.1371/journal.pntd.0013318)
Supplement: S1 Fig — Statistical analysis was performed using the ANOVA test with P value adjusted for sex and age (Padj) for distribution among genotypes and post hoc test for pairwise comparison between genotypes (*P = p value corrected for false discovery rate (FDR)). (PDF) [file pntd.0013318.s001.pdf]

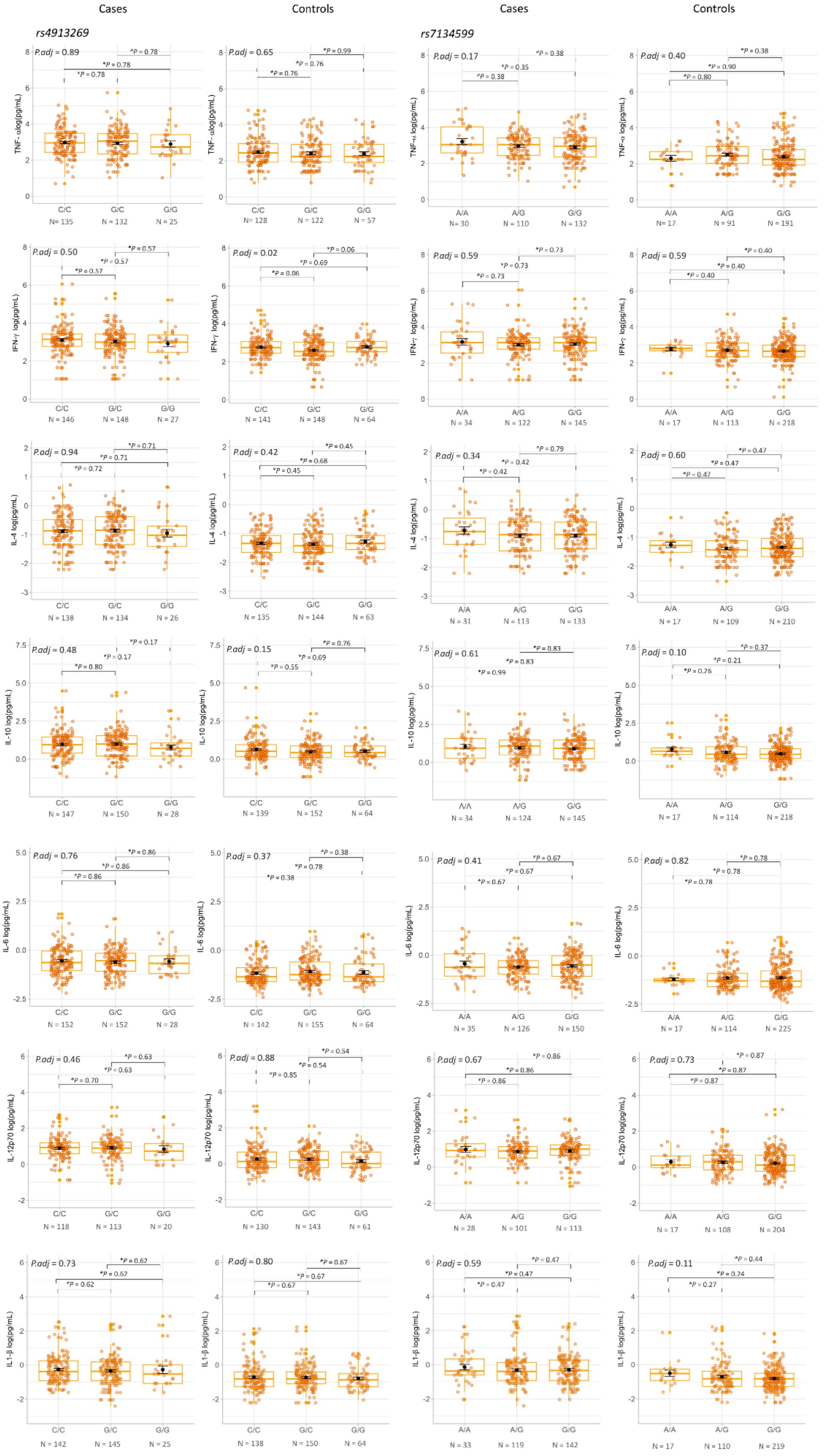
Cases
Controls
Cases
Controls
rs4913269
rs7134599

Supplementary Figure. Analysis of plasma cytokines levels by genotypes of variant rs4913269 and rs7134599 in Cases and Controls. Statistical analysis was performed using the ANOVA test with P value adjusted for sex and age (Padj) for distribution among genotypes and Posthoc test for pairwise comparison between genotypes (*P = p value corrected for false discovery rate (FDR)).
