# Supplementary figures and images for: Genetic analysis of IFNG-AS1 implicates opposite effects to Leishmania guyanensis-cutaneous leishmaniasis: rs4913269 confers protection while rs7134599 enhances susceptibility and correlates with high plasma IL-4 and IL-10 levels
Source: PLoS Negl Trop Dis. 2025 Jul 14;19(7):e0013318. doi: 10.1371/journal.pntd.0013318 (PMC12273940; doi:10.1371/journal.pntd.0013318)

## Healthy Controls

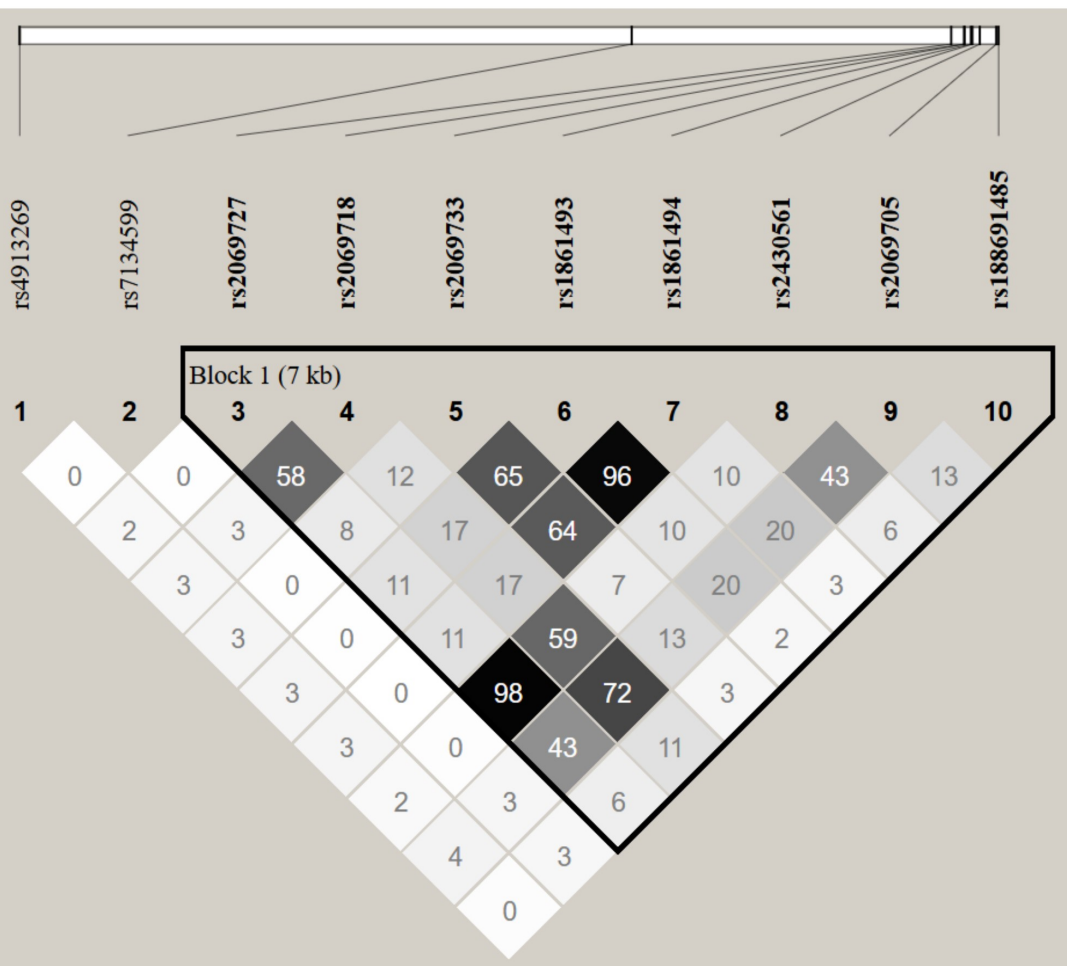

## Patients with *Lg*-CL

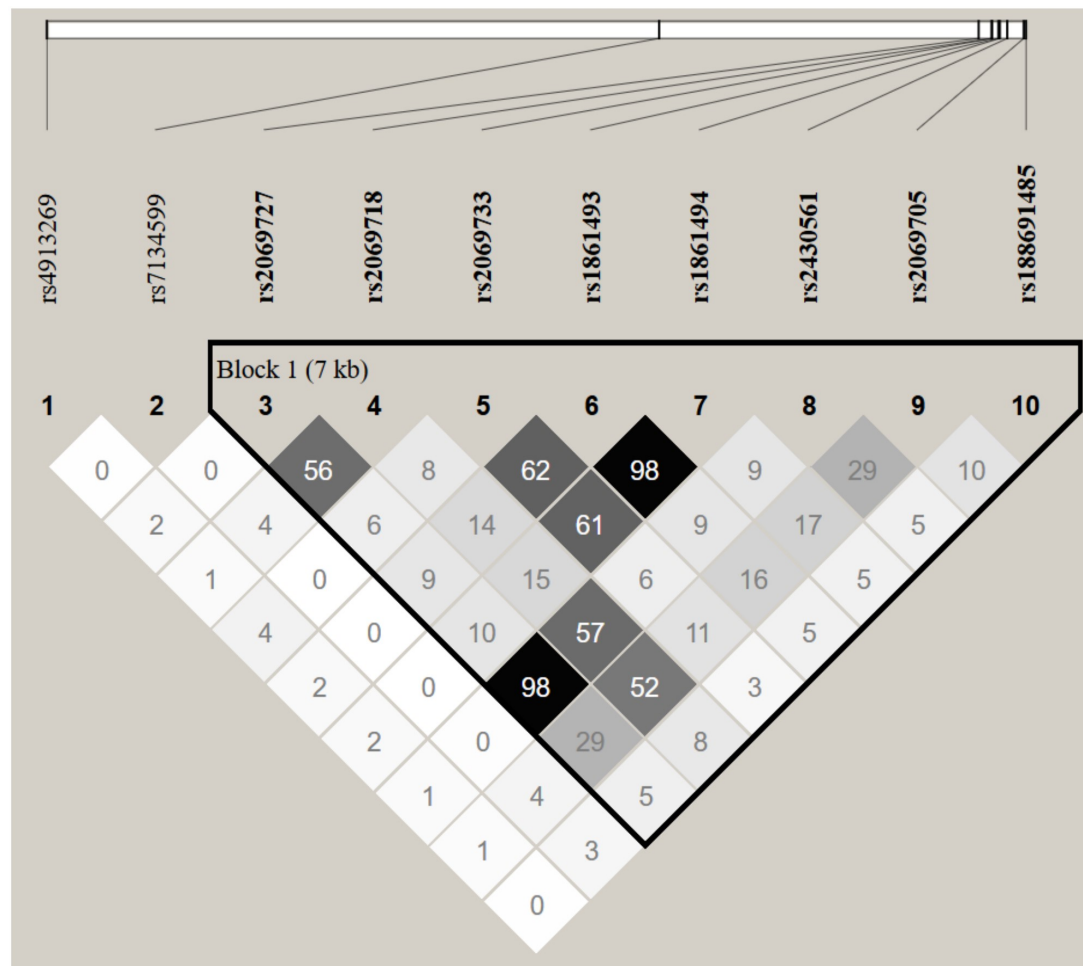

Supplement: S2 Fig — The LD plot was performed using Haploview 4.2 and displays R2 and D’ measures. (PDF) [file pntd.0013318.s002.pdf]
